# Cost-effectiveness of genetic risk-stratified screening for breast cancer in Taiwan

**DOI:** 10.1016/j.breast.2025.104566

**Published:** 2025-08-26

**Authors:** Yu-Chen Hou, Fang-Ju Lin, Yu-Hsuan Joni Shao

**Affiliations:** a Graduate Institute of Biomedical Informatics, College of Medical Science and Technology, Taipei Medical University, Taipei, Taiwan; b Graduate Institute of Clinical Pharmacy & School of Pharmacy, College of Medicine, National Taiwan University, Taipei, Taiwan; c Department of Pharmacy, National Taiwan University Hospital, Taipei, Taiwan; d Clinical Big Data Research Center, Taipei Medical University Hospital, Taipei, Taiwan

**Keywords:** Breast cancer, Risk-stratified screening, PRS, Cost-effectiveness, Markov model, Taiwan

## Abstract

**Background:**

Risk-stratified breast screening has gained international attention, as individualized risk assessments can inform screening initiation, frequency, and whether to screen. In this study, we evaluated the cost-effectiveness of risk-stratified screening based on genetic testing for breast cancer-associated single nucleotide polymorphisms (SNPs) compared to the current age-based screening program in Taiwan.

**Methods:**

A Markov model was used to estimate lifetime health outcomes and costs for 35-year-old Taiwanese women without a family history of breast cancer. The model adopted the healthcare payer's perspective, applied a 3 % annual discount rate, and utilized epidemiological and cost data primarily derived from Taiwanese sources whenever possible. Scenario analyses included various percentile thresholds used to define polygenic risk groups in the risk-stratified screening strategy. Within this strategy, no screening was modeled for women in the low-risk group, while those in the intermediate- and high-risk groups were offered standard biennial mammography, beginning at ages 40 and 35, respectively, and continuing until 69.

**Results:**

Compared to the current age-based mammogram-only screening, polygenic risk scores (PRS)-informed risk-stratified breast cancer screening generated additional costs and quality-adjusted life years (QALYs), with an incremental cost-effectiveness ratio (ICER) of US$75.71/QALY. Scenario analyses using different PRS cutoffs consistently yielded ICERs well below one time Taiwan's gross domestic product per capita per QALY, suggesting cost-effectiveness of genetic risk-stratified screening.

**Conclusion:**

Incorporating polygenic risk into the current breast cancer screening program may improve health outcomes at an acceptable cost. These findings support implementing risk-stratified screening in future policy.

## Highlights

• Risk-stratified breast cancer screening based on PRS was cost-effective.
• Risk stratification into high, intermediate, and low groups enabled tailored screening.
• Sensitivity and scenario analyses supported the cost-effectiveness of genetic risk-stratified screening.

## 1 Background

Breast cancer is the most common cancer type and the leading cause of cancer deaths among women worldwide [1]. The annual report of the Taiwan Cancer Registry for 2021 indicated that breast cancer was the most prevalent type of cancer among Taiwanese women, representing 29.2 % of all cancer diagnoses. The incidence rate of breast cancer in Taiwan has steadily increased over the past 3 decades, almost tripling from 23.53 per 100,000 women in 1995 to 76.15 per 100,000 women in 2021 [2]. An effective breast cancer screening program is crucial, as women diagnosed in advanced stages have significantly lower survival rates compared to those diagnosed at earlier stages [3].

Detecting breast cancer early through screening reduces the number of deaths caused by the disease [4]. However, potential drawbacks include unnecessary tests, overdiagnoses, and excessive treatments [5]. Recognizing the crucial role of early detection in improving breast cancer outcomes, the Taiwan Health Promotion Administration launched the Breast Cancer Screening Program in 2004. Currently, Taiwan employs an age-based screening strategy, offering biennial mammograms free of charge to women aged 45–69 years without a family history of breast cancer, and to women aged 40–69 years with a family history of the disease [6]. Despite the availability of the program, uptake remains suboptimal, with only 40 % participation among eligible women in 2019 [7]. Moreover, the current program's high false-positive rate and "one-size-fits-all" age-based strategy contribute to psychological, medical, and economic burdens, while failing to account for individual risk. A tailored screening approach might improve both effectiveness and efficiency.

Genetic, hormonal, and lifestyle factors significantly contribute to a woman's risk of developing breast cancer. Advances in genomics have led to the identification of more than 300 breast cancer susceptibility loci through genome-wide association studies (GWASs). These loci can be aggregated into a polygenic risk score (PRS), which was shown to account for approximately 20–30 % of the heritable component of breast cancer [8,9]. The PRS offers a promising approach to risk predictions and personalized screening strategies. Based on PRS distributions, individuals can be classified into high-, intermediate-, or low-risk groups, using percentile thresholds derived from GWAS data specific to the population under study.

Risk-stratified screening adopts personalized approaches to detect breast cancer based on an individual's risk level. Such approaches involve intensified screening for people who exceed a specific risk threshold, standard screening for those at average risk, and reduced or no screening for those at lower risk [10]. Such a targeted approach has the potential to maximize benefits of early detection while mitigating harm associated with conventional population-based screening programs [11]. Despite increasing evidence supporting the potential of the PRS as a complement to age-based screening programs, a limited number of countries have adopted this approach [12]. To date, large-scale trials such as PROCAS (Predicting the Risk of Cancer at Screening), WISDOM (Women Informed to Screen Depending on Measures of Risk), and MyPeBS (My Personalized Breast Screening) have been launched to investigate the viability of risk-stratified screening including a breast cancer PRS in population screening methods and exploring strategies to facilitate their clinical use [[13], [14], [15]]. These efforts could provide the necessary foundation for policy makers to make informed decisions regarding the incorporation of the PRS into national age-based screening programs.

Risk-stratified breast cancer screening was demonstrated to be potentially beneficial for women at the population level [[16], [17], [18]]. While BRCA1/2 testing was shown to be cost-effective in high-risk populations, those findings highlight the broader value of incorporating genetic risk information into risk-stratified screening strategies to enhance cancer prevention efforts [[19], [20], [21], [22]]. Reflecting this growing importance, risk-stratified breast cancer screening is increasingly being explored and prioritized in national initiatives, including in Canada [23]. Furthermore, previous studies showed that the cost-effectiveness of traditional age-based breast screening programs could be improved by adopting a PRS-informed risk-stratified screening strategy [[24], [25], [26]]. However, economic evaluations are still needed in Taiwan to assess the cost-effectiveness of adopting PRS-based breast cancer screening, given the distinct characteristics of the disease and the healthcare system. Implementing PRS-stratified screening would require assessing the risk of all women, which could lead to additional costs, but those expenses may be offset by reducing unnecessary screening for women at lower risk of developing breast cancer. Therefore, in this study, we assessed the cost-effectiveness of risk-stratified breast cancer screening based on an individual's PRS-derived risk grouping among Taiwanese women, providing the evidence necessary for its potential implementation to support policymakers in Taiwan.

## 2 Methods

### 2.1 Model design and assumptions

**Fig. 1:**
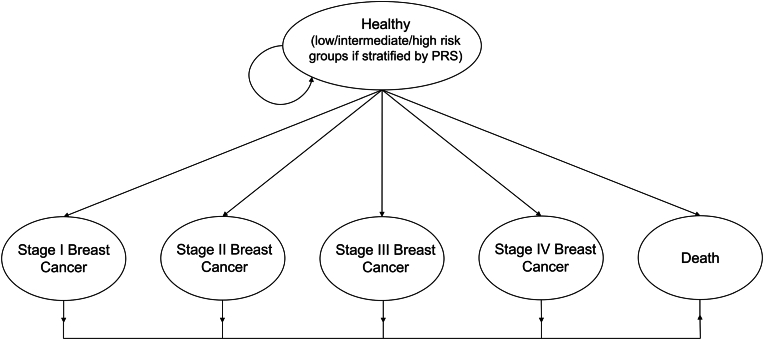
Markov diagram.

A recently developed Markov model (Fig. 1) for breast cancer screening was adopted to evaluate the cost-effectiveness of the proposed risk-stratified screening strategy compared to the current age-based screening program from the perspective of Taiwan's healthcare payer [25]. The model was designed based on a hypothetical population without a family history of breast cancer, with all individuals starting at age 35 years and in a healthy state. Due to the average life expectancy at birth for Taiwanese women being around 84 years, a lifetime horizon of 50 years was used in the Markov model.

**Fig. 2:**
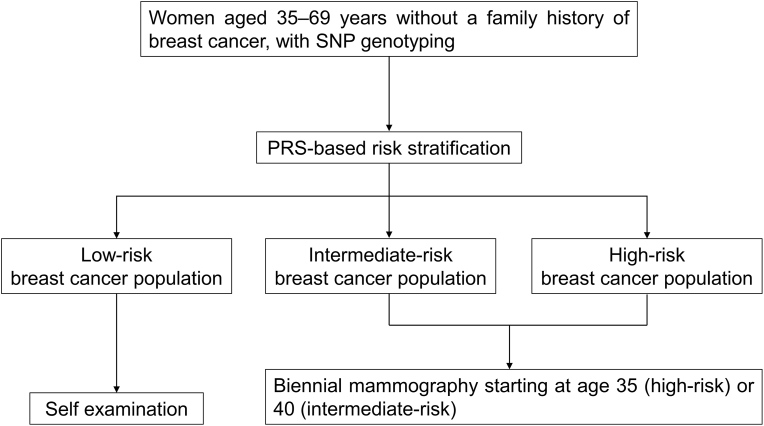
Polygenic risk score (PRS)-stratified screening strategy for breast cancer. The PRS-based risk stratification defined cutoffs as below the 35th percentile for the low-risk group, between the 35th and 95th percentiles for the intermediate-risk group, and above the 95th percentile for the high-risk group.

In the PRS-stratified screening strategy, individuals aged 35 years are to undergo single-nucleotide polymorphism (SNP) genotyping using a SNP array and receive subsequent genetic counseling. Based on their PRS, they will be classified into low-, intermediate-, or high-risk groups. PRS cutoffs are defined as below the 35th percentile for the low-risk group, between the 35th and 95th percentile for the intermediate-risk group, and above the 95th percentile for the high-risk group. The PRS used in this cost-effectiveness analysis was developed specifically for Taiwanese women, and the population distribution between risk groups was expected to be approximately 35 %, 60 %, and 5 % for the low-, intermediate-, and high-risk groups, respectively [27]. In this strategy, individuals in the low-risk group would not undergo regular mammography screening but would be encouraged to perform self-examinations. Those in the intermediate- and high-risk groups would follow the standard screening protocol, which involves biennial mammographic examinations. Specifically, intermediate-risk individuals would begin screening at age 40 years, consistent with Taiwan's current practice of initiating breast cancer screening at that age. High-risk individuals would begin screening at age 35 years, based on research showing that their 10-year breast cancer risk by age 37 years is comparable to that of an average 50-year-old woman [28]. The screening would end at age 69 years to align with Taiwan's current breast cancer screening guidelines (Fig. 2). In contrast, the current age-based screening strategy involves a biennial mammography for all individuals between the ages of 45 and 69 years. Since PRS-informed risk-stratified screening does not incorporate one's family history, restricting the age-based screening group to this population ensures a more-appropriate comparison and avoids potential bias from the inclusion of women with a family history who start screening at 40 years of age.

A Markov model analysis was conducted to estimate costs, life years gained, and quality-adjusted life years (QALYs) gained. The model comprises six states: healthy, stages I to IV breast cancer, and death. Women enter the Markov model in a healthy state. During each 1-year cycle, women can either remain in the healthy state or transition to one of the breast cancer stages (I to IV) or death. The model assumes full participation, with 100 % of the target population engaging in the screening strategy. Furthermore, women diagnosed with breast cancer are assumed not to go into remission but to remain in a diseased state.

### 2.2 Input parameters

All parameters are summarized in Table 1. Parameters were derived from studies conducted in Taiwan or well-established international sources when specific Taiwan data were not available. Age-specific invasive breast cancer incidence rates were calculated using data from the 2021 Taiwan Cancer Registry Annual Report [2] in combination with midyear population by sex and 5-year age groups of Taiwan [29]. Stage distributions of breast cancer for screened and unscreened cases were obtained from a previous study by Lin et al. [30]. They used data from the Mammography Screening database in Taiwan to determine breast cancer stages based on whether a diagnosis was made through mammographic screening or clinical detection. Mammography sensitivity, which accounts for missed cases, was derived from a published Taiwanese article [31]. To illustrate differences in breast cancer incidences between each polygenic risk group, 1.5 × , 1.1 × , and 0.6 × were respectively applied to the high-risk, intermediate-risk, and low-risk groups. These risk group multipliers were determined based on the fact that women in the high-risk group are approximately 1.5 times as likely as the general population to develop breast cancer. Women in the intermediate-risk group are roughly 1.1 times as likely as the general population to develop breast cancer. Furthermore, women in the low-risk group are 0.6 times as likely as the general population to develop breast cancer. These risk differences were based on a previous breast cancer PRS study conducted in Taiwan by Wang et al. (not available to the public until July 2025) [27]. Subsequently, transition probabilities between a healthy state and each disease state were converted based on rates calculated using these parameters (age-specific incidence rates × risk group multiplier (if applicable) × stage-specific proportion × mammography sensitivity). If no screening was conducted during the cycle, the mammography sensitivity was excluded from the transition probability calculation. Stage-specific mortalities were obtained from cancer statistical data published by a cancer center in Taiwan [32]. These stage-specific mortality rates represented aggregated data across all ages. Because age-stratified stage-specific breast cancer mortality estimates were unavailable, we adjusted the base rates using age-specific crude breast cancer mortality rates published by Gender Equality Committee of the Executive Yuan in Taiwan. The crude mortality rates were 5.5 per 100,000 for ages 35–44, 36.4 per 100,000 for ages 45–64, and 63.3 per 100,000 for ages ≥65. We assumed the base rates represented the 45–64 age group and used it as the reference. Age adjustment factors were calculated as the ratio of each group's crude mortality rate to that of the reference group (i.e., age adjustment factor = mortality at age group X/mortality at age group 45–64). This resulted in age adjustment factors of 0.151 for ages 35–44, 1.000 for ages 45–64 (reference), and 1.739 for ages ≥65. These factors were then applied to the base stage-specific mortality rates to derive age-adjusted estimates. Patients could experience natural death between screening cycles [33].

**Table 1:** Input parameters for the cost-effectiveness model.

Variable	Baseline value	Min^a^	Max.^b^	Distribution	Parameters	Reference
Annual discount rate for costs and benefits	0.03	–	–	–		Sun et al. (2018) [37]
Polygenic risk distribution		–	–	–		
Low	0.35					
Intermediate	0.6					
High	0.05					
Breast cancer stage Distribution (Screened/Unscreened)^a^		–	–	–		Lin et al. (2022) [30]
Stage I	0.49/0.35					
Stage II	0.36/0.39					
Stage III	0.13/0.18					
Stage IV	0.02/0.08					
Stage-specific utility values				Beta		Kaur et al. (2022) [35] Yong et al. (2022) [36]
Healthy	1.0	1.0	1.0		–	
Stage I in year 1	0.85	0.75	0.95		α = 40.8; β = 8.2	
Stage II in year 1	0.85	0.75	0.95		α = 40.8; β = 8.2	
Stage III in year 1	0.70	0.60	0.80		α = 55.8; β = 24.9	
Stage IV in year 1	0.52	0.42	0.62		α = 49.3; β = 46.5	
Stage I in later years	0.95	0.85	1.00		α = 29.9; β = 2.6	
Stage II in later years	0.95	0.85	1.00		α = 29.9; β = 2.6	
Stage III in later years	0.85	0.75	0.95		α = 40.8; β = 8.2	
Stage IV in later years	0.60	0.50	0.70		α = 54.7; β = 37.5	
Risk group multiplier		–	–	Normal		Wang et al. (2023) [27]
High	1.5				μ = 1.5; σ = 0.23	
Intermediate	1.1				μ = 1.1; σ = 0.17	
Low	0.6				μ = 0.6; σ = 0.09	
Mammography sensitivity	0.835	–	–	Beta	α = 27.3; β = 6.4	Pan et al. (2014) [31]
Annual transition probabilities						
Age-specific incidence of invasive breast cancer		–	–	–		Midyear Population by Sex and 5-Year Age Group of Taiwan [29], 2021 Taiwan Cancer Registry annual report [2]
35–39	0.000744					
40–44	0.001513					
45–49	0.002383					
50–54	0.002232					
55–59	0.002141					
60–64	0.002262					
65–69	0.002298					
70–74	0.001974					
75–79	0.001666					
80–84	0.001620					
Stage-specific mortality^c^		–	–	–		Koo Foundation Sun Yat-Sen Cancer Center. 2022 Cancer Statistics [32]
Stage I	0.004					
Stage II	0.006					
Stage III	0.025					
Stage IV	0.167					
Age-specific all-cause mortality (excluding breast cancer)		–	–	–		2021 Cause of Death Statistics [33]
35–39	0.000590					
40–44	0.000910					
45–49	0.001409					
50–54	0.002008					
55–59	0.002856					
60–64	0.004460					
65–69	0.006986					
70–74	0.012718					
75–79	0.024427					
80–84	0.046523					
Cost (US$)^d^						
Stage-specific direct medical annual costs				Gamma		Lin et al. (2022) [30]
Stage I	2444.00	1710.8	3177.2		α = 42.7; β = 57.3	
Stage II	3039.56	2127.69	3951.43		α = 42.7; β = 71.2	
Stage III	4678.02	3274.61	6081.43		α = 42.7; β = 109.6	
Stage IV	13,396.20	9377.34	17,415.06		α = 42.7; β = 313.9	
Cost of mammography	44.96	31.47	58.45	Gamma	α = 42.7; β = 1.1	NHI cost
Cost of SNP genotyping	126.40	88.48	164.32	Gamma	α = 42.7; β = 3.0	Local hospitals in Taiwan
Cost of genetic counseling	54.17	37.92	70.42	Gamma	α = 42.7; β = 1.3	Local hospitals in Taiwan

US$: United States dollars; NHI: National Health Insurance; SNP: single-nucleotide polymorphism; α: shape parameter; β: rate parameter; μ: mean; σ: standard deviation.

a “Screened” refers to individuals who undergo mammography screening; “Unscreened” refers to individuals who do not undergo mammography screening.

b Minimum and maximum values represent ranges used in the one-way sensitivity analysis. Distributions were used for a probabilistic sensitivity analysis (PSA).

c Stage-specific mortality was derived from aggregated data and assumed to reflect ages 45–64. Age adjustment factors (0.151 for ages 35–44 and 1.739 for ages ≥65) were applied based on Taiwanese age-specific crude breast cancer mortality. See Methods.

d US$ at exchange on 2021/12/30 (1 US$ = 27.69 New Taiwan Dollar).

From the payer's perspective, direct medical costs by breast cancer stage was derived from Lin et al. [30]. The cost of mammography was obtained from the reimbursement price of the National Health Insurance (NHI) in Taiwan. As SNP genotyping and genetic counseling are not reimbursed by Taiwan's NHI currently, we obtained those costs through out-of-pocket expenses from hospitals in Taiwan. All monetary values in this study are reported in United States dollars (US$). To ensure consistency, costs were adjusted to 2021 US$ using the consumer price index (CPI) to account for inflation, and local currency values were converted to US$ using the 2021 exchange rate [34]. The model outcomes were life years gained and QALYs gained. QALY is a measurement that reflects both the length of life and the health-related quality of life. It is calculated by multiplying the time spent in each health state by an appropriate utility score. Stage-specific health utility scores used in this study were derived from a systematic review of utility values in breast cancer and the Canadian OncoSim-Breast Cancer model [35,36], as those parameter estimates were unavailable in Taiwan. A tunnel-state structure was applied to reflect different utilities in the first year after diagnosis versus subsequent years. The discount rate used for costs and health outcomes was 3 % in this model [37].

### 2.3 Uncertainty analyses

To verify the robustness of our results, we performed one-way sensitivity analyses to explore factors that influenced the results. When other parameters remain unchanged, by changing the value of a certain influencing factor within a predetermined range, the degree of influence of that factor was used to examine the stability of the model. Factors included in the one-way sensitivity analyses were as follows: the risk group multiplier, mammography sensitivity, stage-specific direct medical cost, cost of SNP genotyping, cost of genetic counseling, and stage-specific utility values. All values were set to low and high values of 20 % less or more than the base case value, respectively, as the possible range of variation. A tornado diagram was used to demonstrate the influencing factors of the incremental cost-effectiveness ratio (ICER) in the breast cancer screening strategy. To test the sensitivity of the results to changes in discounting assumptions, additional analyses were conducted using annual discount rates of 0 %, 1 %, and 5 %, in comparison to the base-case assumption of 3 %. This range reflects international variability in discounting practices and allows for a more comprehensive understanding of the impact of discounting on model outcomes.

A probabilistic sensitivity analysis (PSA) was performed to examine parameter uncertainty and estimate a cost-effectiveness acceptability curve using a Monte Carlo simulation with 10,000 iterations. The PSA was conducted on cost parameters (gamma distribution) and utilities (beta distribution). To account for the uncertainty in cost and utility values, cost values were assumed to vary within a ±30 % range, while utility values varied within a ±0.1 range. In addition, the PSA incorporated the uncertainty of risk group multipliers, modeled as normal distributions with standard deviations derived from a ±30 % range. Mammography sensitivity was also included using a beta distribution, with values varying within ±15 % of the base-case estimate. The willingness-to-pay (WTP) threshold represents an estimate of what an individual is willing to pay for a health benefit, specifically to gain 1 QALY. After generating 10,000 estimates of incremental costs and incremental effects through the PSA, we then plotted a cost-effectiveness acceptability curve to display the proportion of simulations in which risk-stratified screening was considered cost-effective at various WTP thresholds.

Given that the cutoffs to stratify polygenic risk groups are somewhat arbitrary, we evaluated the impacts of adopting different cutoff values for the risk groups in a scenario analysis. For the high-risk group, cutoff points at the fifth to the 10th percentiles were explored, while for the low-risk group, cutoff points at the 30th to 35th percentiles were explored. These ranges were covered by three separate scenarios: (1) 30th percentile low−60th percentile intermediate–10th percentile high (30 L-60I-10H), (2) 35th percentile low–55th percentile intermediate–10th percentile high (35 L-55I-10H), and (3) 30th percentile low–65th percentile intermediate–5th percentile high (30 L-65I-5H), all compared to the base case scenario of the 35th percentile low–60th percentile intermediate–5th percentile high (35 L-60I-5H). Other scenario analyses were performed by varying the risk-stratified screening start age of the high-risk breast cancer population (35 or 40 years) and stop age of both the high- and intermediate-risk breast cancer populations (69 or 74 years old). Moreover, a scenario analysis was performed by simultaneously varying the start and stop ages of age-based screening (45–69 years or 40–74 years). In this scenario, the stop age for risk-stratified screening was also extended to 74 years to align with the modified upper limit in the age-based strategy. Another scenario analysis was conducted in which it was assumed that individuals would undergo SNP genotyping and genetic counseling at 40 years of age. In this scenario analysis, the risk-stratified screening start age of the high-risk breast cancer population would also be 40 years. Additionally, since proportions of the population with PRS below the 35th percentile, within the 60th to 95th percentile, and above the 95th percentile for the low-, intermediate and high-risk groups were based on data from where the PRS was developed, these proportions could differ in real-world settings. To address this, we conducted a scenario analysis assuming that the population proportions for the low-, intermediate-, and high-risk groups were 40 %, 50 %, and 10 %, respectively.

## 3 Results

### 3.1 Base-case analysis

During a time frame of 50 years, the cost-effectiveness model estimated 53.98 cases of breast cancer per 1000 women in the risk-stratified screening arm, compared to 56.63 cases in the age-based mammogram screening arm. The risk stratified screening strategy cost US$2478.52 per woman in the base-case scenario, resulting in an incremental cost of US$74.75 compared to the current mammogram screening strategy. The incremental gains in life years and QALYs per woman with risk-stratified screening were 0.9859 and 0.9874, respectively. Overall, risk-stratified screening resulted in more-favorable effects, including life years and QALYs, than age-based mammogram screening, but was associated with higher costs. The incremental cost-effectiveness ratio (ICER) for the base case analysis was US$75.71/QALY gained (Table 2).

**Table 2:** Costs and health outcomes for comparison of two screening strategies in the base-case analysis.

Strategy	Lifetime costs per case (US$)	Life years	Quality-adjusted life years (QALYs)	Incremental calculations (risk-stratified – current mammogram)			
				Costs	Life years	QALYs	ICER (US$/QALY)
Current mammogram screening	2403.77	24.29	24.24	–	–	–	–
PRS-stratified screening	2478.52	25.28	25.23	74.75	0.9859	0.9874	75.71

ICER: incremental cost-effectiveness ratio; PRS: polygenic risk score.

### 3.2 Scenario analysis

In a scenario analysis, various percentile cutoffs for risk groups in the risk-stratified screening strategy were examined, using splits of 30L-60I-10H, 35L-55I-10H, and 30L-65I-5H, resulting in ICERs of US$185.88, 119.56, and 141.84/QALY, respectively (Table 3). In one scenario analysis, beginning screening at an older age (40 years) for the high-risk group in the risk-stratified screening strategy would contribute to a slightly lower QALY gain and a smaller increase in costs compared to the base-case scenario, resulting in an ICER of US$73.46/QALY. Stopping screening at an older age (74 years) for both the high- and intermediate-risk groups in the risk-stratified screening strategy would lead to a modest increase in QALY gains and a greater increase in costs compared to the base-case scenario, resulting in an ICER of US$90.68/QALY. Moreover, simultaneously varying the start and stop ages of age-based screening (from 45 to 69 to 40–74 years) would substantially reduce the increase in costs while slightly lowering the QALY gains compared to the base-case scenario, resulting in an ICER of US$17.95/QALY. In another scenario analysis, undergoing SNP genotyping and genetic counseling at a later age of 40 years, combined with the risk-stratified screening start age of 40 years for both the high- and intermediate-risk groups, would result in slightly lower QALY gains and a smaller increase in costs compared to the base-case scenario, yielding an ICER of US$67.12/QALY. In a different scenario, where the proportions of the population with a PRS below the 35th percentile, within the 60th to 95th percentiles, and above the 95th percentile for low-, intermediate and high-risk groups were 40 %, 50 % and 10 %, respectively, the ICER would decrease to US$53.48/QALY.

**Table 3:** Modeled cost-effectiveness of risk-stratified screening within different scenarios compared to age-based mammogram screening.

Scenario		Incremental calculations (risk-stratified - current)			
		Costs	Life years	QALYs	ICER (US$/QALY)
Base case		74.75	0.9859	0.9874	75.71
Risk group cutoffs	35th low-60th int-5th high (base case)	74.75	0.9859	0.9874	75.71
	30th low-60th int-10th high	182.91	0.9845	0.9840	185.88
	35th low-55th int-10th high	117.86	0.9852	0.9857	119.56
	30th low-65th int-5th high	139.80	0.9853	0.9856	141.84
Screening start age of high-risk group in risk-stratified screening	35 years (base case)	74.75	0.9859	0.9874	75.71
	40 years	72.51	0.9856	0.9870	73.46
Screening stop age in risk-stratified screening	69 years (base case)	74.75	0.9859	0.9874	75.71
	74 years	89.60	0.9864	0.9881	90.68
Screening start and stop age in age-based screening	45–69 years (base case)	74.75	0.9859	0.9874	75.71
	40–74 years	17.63	0.9818	0.9821	17.95
SNP genotyping start age	35 years (base case)	74.75	0.9859	0.9874	75.71
	40 years	65.24	0.9724	0.9721	67.12
Population proportions for risk groups	35 %, 60 %, 5 % for low, int, high (base case)	74.75	0.9859	0.9874	75.71
	40 %, 50 %, 10 % for low, int, high	52.81	0.9858	0.9875	53.48

QALY: quality-adjusted life year; ICER: incremental cost-effectiveness ratio; int: intermediate; SNP: single-nucleotide polymorphism.

### 3.3 Sensitivity analysis

**Fig. 3:**
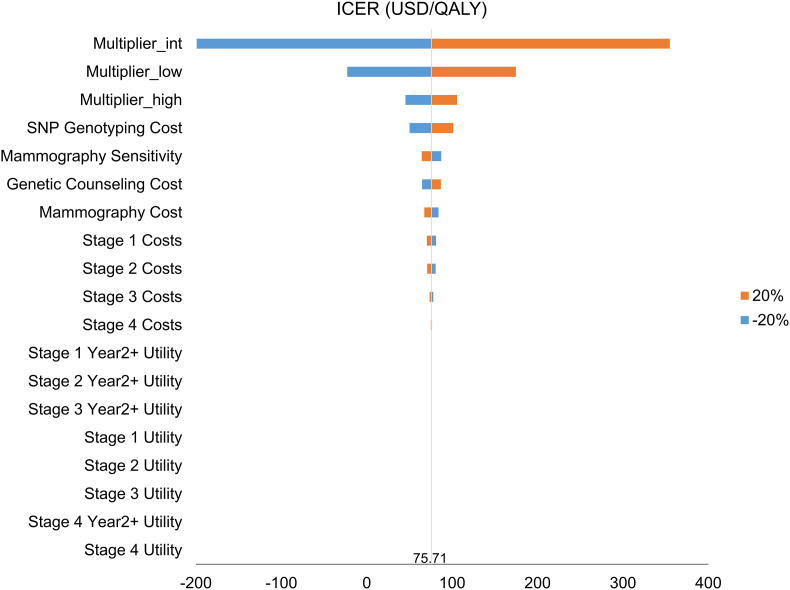
Tornado diagram of a one-way sensitivity analysis. The intermediate-risk group multiplier (multiplier_int), which represents differences in breast cancer incidences for the intermediate-risk group compared to the general population, was the most significant driver of cost-effectiveness for breast cancer screening strategies. This was followed by the low-risk group multiplier (multiplier_low) and the high-risk group multiplier (multiplier_high). Parameters are ranked by their impact on the ICER. USD, United States dollars; QALY, quality-adjusted life years.

To assess impacts of the parameters on the ICERs, both one-way and probabilistic sensitivity analyses were conducted. In the one-way sensitivity analysis, which is illustrated in a tornado chart (Fig. 3), the intermediate-risk group multiplier was identified as the most important driver of cost-effectiveness for breast cancer screening strategies, followed by the low-risk group multiplier and high-risk group multiplier. The cost of SNP genotyping, sensitivity of mammography, and cost of genetic counseling were also key determinants of the ICER. ICERs become lower when either the intermediate-, low-, or high-risk multiplier was calculated using the lower limit (80 %) of the parameter. In the case of the intermediate- and low-risk multipliers, this led to negative ICERs, indicating potential dominance of the risk-stratified screening strategy. Although an exact cost-effectiveness threshold has not been established in Taiwan, the ICER value of less than 1 gross domestic product (GDP) per capita per QALY suggests cost-effectiveness according to criteria proposed by the WHO [38]. Taiwan has a GDP per capita exceeding US$30,000. The tornado diagram indicates that across all intervals examined in the one-way sensitivity analysis, the risk-stratified screening strategy was considered cost-effective compared to the age-based mammogram screening strategy, given a WTP US$30,000/QALY. The ICER rose from US$75.71/QALY at the base-case discount rate of 3 % to US$132.80/QALY at 5 %, while decreasing to –US$34.25/QALY and –US$125.42/QALY under 1 % and 0 % discounting, respectively. This suggests that the cost-effectiveness of the intervention is sensitive to the discount rate; however, as all values remain well below the WTP threshold of US$30,000/QALY, the intervention remains cost-effective or even dominant.

**Fig. 4:**
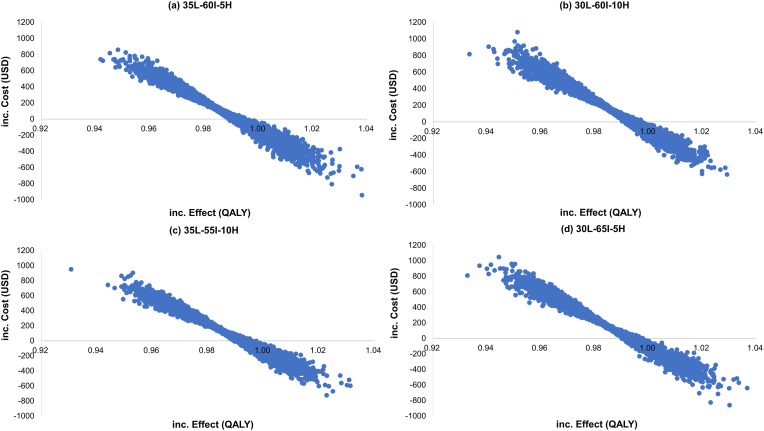
Cost-effectiveness planes for probabilistic sensitivity analysis for the base case and three scenarios in the scenario analysis. (a) 35L-60I-5H, (b) 30L-60I-10H, (c) 35L-55I-10H, (d) 30L-65I-5H.

**Fig. 5:**
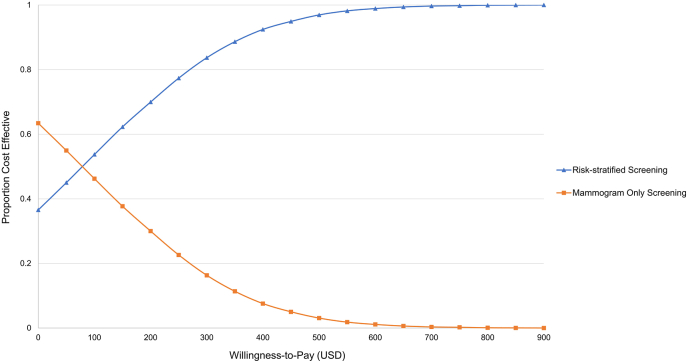
Cost effectiveness acceptability curve from the probabilistic sensitivity analysis for the base-case scenario.

Fig. 4a presents the incremental cost-effectiveness plane from the PSA on the baseline scenario. In the Monte Carlo simulation, all iterations were well below a WTP threshold of US$30,000/QALY gained. This indicated that the PSA suggests that risk-stratified screening could be more cost-effective than the current age-based mammogram screening strategy. Notably, some ICERs crossed from positive to negative, indicating that risk-stratified screening may dominate age-based screening, with incremental costs <0 and incremental effects >0. The cost-effectiveness acceptability curve showed a probability of 70 % of the risk-stratified screening strategy being cost-effective at a US$200/QALY willingness to pay (Fig. 5). Furthermore, 100 % of the iterations became cost-effective at a minimum threshold of US$900/QALY, with 37 % already cost-effective at a WTP of US$1/QALY. On the other hand, among PSAs done on the three additional scenarios with different risk group cutoffs (Fig. 4b–d), all iterations were also below the WTP threshold of US$30,000/QALY gained. The 30L-60I-10H scenario showed a lower incremental effect and higher incremental cost compared to the other scenarios.

## 4 Discussion

Our study demonstrates that risk-stratified breast cancer screening based on the PRS would be highly cost-effective in Taiwan, with an ICER of US$75.71/QALY, far below the WHO-recommended cost-effectiveness threshold of 1 × GDP per capita (more than US$30,000 in Taiwan) per QALY gained [38]. These findings provide evidence that integrating PRSs into breast cancer screening programs could be feasibly implemented in Taiwan. Advising individuals with a screening strategy based on their PRS-predicted risk would result in QALY gains with an acceptable additional cost. To our knowledge, this is the first study to evaluate the cost-effectiveness of risk-stratified breast cancer screening based on the PRS in an East Asian population, addressing critical gaps in previous research that focused on Western and Southeast Asian countries. Our results suggest that a PRS-based breast cancer screening strategy could offer a more personalized and cost-effective approach than Taiwan's current age-based mammogram screening program.

To contextualize our model assumptions, we note that although age-specific breast cancer incidence rates from Taiwan's cancer registry in 2021 were used as model inputs, the broader trend of rising incidence in Taiwan merits further consideration. While the incidence rate of breast cancer has nearly tripled over the past three decades, this increase is unlikely to be solely attributable to enhanced detection through screening (i.e., a screening artifact). A recent population-based study reported only a modest rise in early-stage breast cancer diagnoses, suggesting a limited role of screening in inflating incidence rate [39]. This implies that the growing disease burden likely reflects a genuine increase in breast cancer risk, driven by evolving reproductive patterns and lifestyle factors. Accordingly, our projections of risk-stratified screening benefits are grounded in realistic assumptions and remain relevant to current public health needs.

While risk-stratified screening based on PRSs is still in the initial stages of implementation in most countries, prior cost-effectiveness analyses conducted in the UK and Singapore demonstrated its potential benefits [24,25]. However, due to differences in genetic risk profiles, healthcare systems, and cost structures across populations, country-specific evaluations are essential before policy adoption. Our findings align with previous studies, reinforcing the economic advantage of PRS integration and highlighting its potential to improve breast cancer screening outcomes. By implementing a PRS-based screening strategy, Taiwan could serve as a model for other East Asian countries, paving the way for a more effective and sustainable approach to breast cancer prevention.

More QALYs are gained with risk-stratified screening compared to standard age-based screening, with a per-individual difference of 0.9874. This suggests that risk-stratified screening provides a modest improvement over age-based screening, but does not lead to a substantial change in the stage distribution of breast cancer cases. Although the QALY gain is modest, the result is favorable for two reasons. First, no screening was modeled for individuals in the low-risk group under the risk-stratified screening strategy. Second, it indicates that transitioning from age-based screening to risk-stratified screening does not introduce additional harm. The findings also indicate that there are no considerable differences in cancer survival between the two strategies, as QALY is related to survival times and quality of life, the latter of which is related to cancer staging. These data are comparable to estimates from a study in another Asian country, Singapore, suggesting that our model is reasonably robust [25]. To further validate our model, we compared the cumulative cancer risk with published data. Our cost-effectiveness model projected approximately 54 cases of breast cancer per 1000 women in the risk-stratified screening arm over the age range of 35–84 years. In comparison, the age-based mammogram screening arm estimated 57 cases per 1000 women. According to a previous study, women in Taiwan have a cumulative risk of developing breast cancer ranging from 4 % to 10 %, which translates to 40 to 100 cases per 1000 women. This further indicates that our model exhibits a substantial degree of robustness [40].

Unlike age-based mammogram screening, risk-stratified screening customizes the process by adjusting screening intervals, selecting appropriate screening modalities such as mammography alone or in combination with ultrasound or magnetic resonance imaging (MRI), and determining the optimal beginning and stopping ages for screening [10]. However, limited understanding exists regarding whether the pre-symptomatic detection window for breast cancer varies by risk level, and no established guidelines dictate adjustments to screening intervals based on risk. This study modeled an approach where lower-risk individuals forgo screening, while those above a specified risk threshold receive standard screening. Additionally, this study proposed adjusting the breast cancer screening age based on risk levels, recommending a starting age of 35 years for the high-risk group (PRS above the 95th percentile) and 40 years for the intermediate-risk group (PRS between the 35th and 95th percentiles).

In this study, we conducted scenario analyses to assess whether PRS-informed risk-stratified screening can remain cost-effective under different conditions, providing insights for future breast cancer screening policies. In the base case analysis, the screening start age for the high-risk group in the risk-stratified strategy was set to 35 years. For the scenario analysis, the starting age was adjusted to 40 years to align with Taiwan's current practice of initiating breast cancer screening at 40 years for women with a family history. As the ICER for the scenario analysis was lower than that of the base case, setting the screening start age at 40 years for high-risk individuals may be a more cost-effective option for future policy considerations. In the base case analysis, the screening stop age was set to 69 years in the risk-stratified strategy, aligning with Taiwan's current breast cancer screening strategy. However, in the scenario analysis, the stop age was extended to 74 years to incorporate recommendations from the US Preventive Services Task Force (USPSTF). This adjustment resulted in an ICER of US$90.68/QALY, which was higher than in the base case, indicating that this strategy may be less cost-effective. Expanding the screening age range in the age-based strategy from 45 to 69 to 40–74 years resulted in a lower ICER for the risk-based screening strategy, suggesting that PRS-informed risk-stratified screening remains highly cost-effective even when compared to more-intensive age-based screening. Additionally, the scenario analysis set the start age for SNP genotyping at 40 years to evaluate whether initiating risk stratification and screening at a later age would improve the cost-effectiveness. The ICER in this scenario analysis was lower than in the base case (US$67.12 vs. 75.71/QALY), suggesting that a starting age of 40 years for SNP genotyping should be considered when implementing a risk-stratified breast cancer screening strategy. Regarding different risk group cutoffs, the scenario analysis produced a maximum ICER of US$185.88/QALY in the 30th low-60th intermediate-10th high scenario. This indicates that risk-stratified screening remained cost-effective across various cutoff points when the WTP per QALY was set at 1 × GDP per capita. Overall, the scenario analyses emphasized the flexibility of risk-stratified screening, enabling policymakers to refine strategies based on local population data.

Risk-stratified screening, combined with personalized counseling, enhances understanding and awareness of one's genetic predisposition, encouraging high-risk individuals to engage in preventive measures like receiving regular mammograms. By increasing risk awareness through the PRS, this approach reinforces the importance of screening and significantly improves adherence rates [41,42]. Additionally, the Polygenic Risk Score Task Force of the International Common Disease Alliance emphasized the lack of economic evidence of PRS utilization and recommended prioritizing research in this area [43]. This study demonstrated that a PRS-informed risk-stratified screening strategy would modestly improve both clinical benefits and cost-effectiveness compared to an age-based mammogram-only approach. However, it also revealed that risk-stratified screening incurs slightly higher costs than age-based screening. It is important to note that this finding differs somewhat from previous economic studies [24,25], which suggested that risk-stratified screening can be cost-saving in certain scenarios. This discrepancy likely stems from the use of more-conservative risk group multipliers in our model and the relatively lower cost of mammograms in Taiwan. Overall, this study offers a framework for policymakers to integrate genetic risk-stratified approaches into existing screening programs.

Our study did not model screening for individuals in the low-risk group while applying standard screening to the moderate- and high-risk groups. Within Taiwan's current age-based breast cancer screening program, a more-practical approach to gradually implementing risk-based screening could be to first target a subset of the population exceeding a defined risk threshold. However, physicians may be reluctant to completely eliminate screening for patients at much lower risk [44]. Furthermore, low-risk women may resist reduced screening, as they have come to find regular screening reassuring, and a reduction in screening could be perceived as service rationing [45]. Therefore, it is crucial to recognize and address the challenges involved in implementing a risk-based screening program. Key considerations include effectively communicating with both women and healthcare professionals to ensure acceptance of genetic testing for risk stratification and screening eligibility. First, healthcare providers must receive training to accurately assess breast cancer risks, provide prevention guidance based on risk levels, and manage primary care workflows, including DNA extraction from blood or saliva samples. Second, collaboration with specialists, such as oncologists and breast surgeons, is necessary for further genetic testing and the initiation of risk-reduction approaches. Third, equitable access to risk-stratified screening programs is crucial for maintaining fairness in healthcare delivery. Fourth, regulatory approvals are necessary to implement genetic-based risk stratification within clinical practice. Finally, evaluating the potential psychological impacts of risk-stratified screening is critical to address concerns related to genetic risk disclosure and its implications [24,46]. While these barriers present challenges to implementation, general practitioners in the UK have expressed optimism about the potential impact of risk-stratified breast cancer screening incorporating a PRS, highlighting its feasibility and potential benefits in clinical practice [44]. Furthermore, survey-based studies suggest that women would be willing to undergo genetic profiling to determine the appropriate frequency of screening [47,48]. On a more-positive note, Taiwan's highly accessible healthcare system, which provides comprehensive, coordinated, and continuous care [49], creates a strong foundation for the successful implementation of risk-stratified breast cancer screening. In summary, public involvement is essential in decisions regarding modifications to the proposed risk-stratified screening program. Ensuring these decisions are evidence-based and transparently communicating both the benefits and potential harms of the program are key to its successful implementation.

This study is subject to several limitations. First, this study relied on model-based estimates based on assumptions, which might not fully reflect real-world conditions. For example, the model assumed 100 % attendance for SNP genotyping and perfect adherence to breast cancer screening and follow-ups, whereas actual screening participation is lower. In Taiwan, the adherence rate to breast cancer screening was approximately 40 % in 2019 [7]. Compared to other Asian countries, 39.6 % of Singaporean women aged 50–69 years had undergone breast cancer screening within 2 years leading up to 2010 [25]. In terms of cost assumptions, the model applied stage-specific average annual costs based on lifetime medical expenditure data from a Taiwanese population-based study [30]. While this simplification does not capture time-varying cost patterns such as initial treatment peaks or end-of-life care, it reflects real-world stage-specific cost differences and facilitates model tractability. Second, the sensitivity of mammography varies over time, which can introduce uncertainty in the model. Pan et al. reported that the sensitivity of mammography screening ranged from 79.6 % to 87.0 % after 2006 [31]. To address this, the mammography sensitivity parameter was set to 83.5 % in the base case of our model, with a ±20 % variation applied in the one-way sensitivity analysis. Third, the PRS used to stratify individuals in the risk-stratified screening group was derived from a population that included both women with and those without a family history of breast cancer, whereas the age-based screening group in our model was limited to women without a family history. This inconsistency in the population source may have led to a slight overestimation of the benefits of the risk-stratified screening strategy. However, the proportion of individuals with a family history in the PRS derivation dataset is relatively low, and thus the potential impact on the model's results is likely to be minimal. Fourth, our model relied solely on observed breast cancer screening outcomes, including stages I–IV, rather than incorporating the natural history of the disease, which may include a detectable preclinical phase. However, this limitation applies equally to both risk-stratified and age-based screening approaches, likely balancing their impact, particularly as our focus is on comparing the cost-benefit differences between the two strategies. Furthermore, the model did not include health states after breast cancer treatment, such as the post-cancer state after remission. However, this omission is not expected to significantly influence the conclusions of this study, as we anticipate that the number of breast cancers detected by screening will serve as a proxy for these longer-term results. Fifth, our model did not account for potential harms associated with screening, including overdiagnosis, false-positive results, recall examinations, and related psychological impacts. While these factors are important in real-world implementation, they were beyond the scope of our current model structure. Nevertheless, recent evidence suggests that risk-stratified screening may reduce overdiagnosis and improve the benefit-to-harm balance compared to age-based approaches [24]. Finally, since the age-based screening modeled in this analysis included only women without a family history of breast cancer, the cost-effectiveness of risk-stratified screening for women with a family history remains unknown. Despite these limitations, our study highlights the cost-effectiveness of the current age-based mammogram-only strategy that could be improved by adopting a PRS-informed risk stratified screening strategy for women without a family history of breast cancer.

## 5 Conclusion

Our research shows that for women without a family history of breast cancer, a risk-stratified screening strategy—where low-risk individuals forgo screening, while intermediate- and high-risk individuals undergo biennial mammography beginning at ages 40 and 35 years, respectively—proved to be more cost-effective than an age-based mammogram-only program. PRS-informed risk stratification would lead to higher QALYs at an affordable cost. While future studies should address data robustness, model limitations, and the capacity implications of introducing risk-stratified breast cancer screening, this study provides valuable evidence supporting the feasibility of a risk-stratified breast cancer screening approach for policymakers, healthcare professionals, the public, and the scientific community.

## CRediT authorship contribution statement

**Yu-Chen Hou:** Writing – review & editing, Writing – original draft, Methodology, Formal analysis, Data curation, Conceptualization. **Fang-Ju Lin:** Writing – review & editing, Validation, Supervision, Methodology. **Yu-Hsuan Joni Shao:** Writing – review & editing, Supervision, Project administration, Conceptualization.

## Ethics approval and consent to participate

Ethical approval was not required for this study, as it is a cost-effectiveness analysis based on a simulation model. All input parameters were derived from publicly available data in previously published studies. No primary data collection involving human participants or personal information was conducted.

## Consent for publication

Not applicable.

## Data availability

No data were collected for this study. All data used in the economic model are taken from the research literature and referenced in the manuscript.

## Funding

This work was not supported by any funding source.

## Declaration of competing interest

The authors declare that they have no known competing financial interests or personal relationships that could have appeared to influence the work reported in this paper.
